# Non-pharmacologic Approaches in Preoperative Anxiety, a Comprehensive Review

**DOI:** 10.3389/fpubh.2022.854673

**Published:** 2022-04-11

**Authors:** Rulin Wang, Xin Huang, Yuan Wang, Masod Akbari

**Affiliations:** ^1^Medical College, Xijing University, Xi'an, China; ^2^Department of Psychiatry, Wuhan Fourth Hospital, Puai Hospital, Tongji Medical College, Huazhong University of Science and Technology, Wuhan, China; ^3^Research Center for Psychiatric Diseases, Tehran University of Medical Sciences, Tehran, Iran

**Keywords:** preoperative anxiety, non-pharmacological, cognitive-behavioral therapy, aromatherapy, relaxation

## Abstract

During the pre-operation period, surgical candidates experience situations that stimulate psychological anxiety leading to stress during and after surgery which is known as preoperative anxiety. This condition can cause psychological and physiological adverse effects on both children and adults. Due to the high prevalence and adverse effects of preoperative anxiety, different treatments have been evaluated including pharmacological and non-pharmacological approaches. As pharmacological treatments may cause adverse effects such as breathing problems, drowsiness, interfering with anesthetic drugs, and prolonged recovery, non-pharmacological interventions are becoming more popular. These methods include cognitive-behavioral therapy, music therapy, pre-op preparation video, aromatherapy, hypnosis, guided imagery relaxation therapy, and massage. In this study, the most popular non-pharmacological approaches to preoperative anxiety are reviewed focusing on more recent evidence provided by clinical studies. The reviewed clinical evidence on the mentioned methods shows the efficacy of non-pharmacological interventions for the treatment of preoperative anxiety, so they can be used in patients of different ages and types of disease and surgery.

## Introduction

Anxiety is defined as an unpleasant sense associated with fear, tension, and nervousness (1). Surgery as a major trauma can cause anxiety. During the period of pre-operation, surgical candidates experience situations that stimulate psychological anxiety leading to stress during and after surgery. The anxiety secondary to disease, hospitalization, and planned surgery is called preoperative anxiety (2, 3). The most common cause of preoperative anxiety is waiting for surgery, concern about the operation results, separation from family, postoperative pain anticipation, loss of independence, and fear of surgery and death (4).

The prevalence of preoperative anxiety varies widely and it has been reported to range from 40 to 60%among young children patients and 11–80% among adult ones (4–6). In a study, 23.99% of patients experienced severe preoperative anxiety (4). Different causes are proposed for preoperative anxiety such as the fear of the unknown, fear of being sick, and fear of death (7). Various factors are associated with preoperative anxiety. These factors are classified as sociodemographic factors, psychosocial variables, and surgery and anesthesia-related factors such as previous surgical experience, having information about the surgical process, and anesthesia (4, 7). Age is a protective factor of preoperative anxiety as each 1 year increase in age reduces five percent of the chance of preoperative anxiety. Females are at higher risk and levels of anxiety than men and educated persons experience higher levels of anxiety (8). Married patients have greater emotional supports so they experience lower anxiety levels (9). The significance of the surgery is associated with anxiety as higher levels of anxiety are reported in patients who had a greater surgical procedure (10). History of cancer is an important risk factor for preoperative anxiety (4). Previous psychiatric diseases, such as depression and anxiety may influence the extent of preoperative anxiety (11, 12).

Preoperative anxiety can cause psychological and physiological adverse effects on both children and adults. Also, it can interfere with the process of surgery and can put patients in danger during the surgical process (13). Maladaptive behaviors, emergence delirium, and preoperative anxiety are common among children undergoing surgery and these phenomena are related as maladaptive behavioral responses like sleep and eating disturbances and enuresis are common adverse events among children with preoperative anxiety (6, 14–16). A 10 point increase in the state anxiety scores in children may result in a 12.5% increase in the probability of the new-onset maladaptive behavior happening after the operation (16).

The increased need for postoperative analgesics, prolonged hospital stay, and recovery are common among adults (17, 18). Besides, it can trigger autonomic and endocrine systems which cause hemodynamic instability (19). Moreover, severe preoperative anxiety is associated with impaired wound healing and postoperative complications like nausea, vomiting, and pain (20–22). There is a significant inverse relationship between anxiety and recovery and effectiveness of anesthesia (23).

Preoperative anxiety is a matter of concern for many health professionals including anesthesiologists and surgeons, and nurses at the recovery unit, ICU, and ward. It is considered a major morbidity factor during and after the surgical process (24, 25). It is also known as a financial burden on the healthcare system (26). The mentioned costs are considered to be attributed to the prolonged recovery and hospital stay and increased need for anesthetic and analgesic drugs (27).

Due to the high prevalence and adverse effects of preoperative anxiety, treatment is necessary. Till now, two types of interventions for preoperative anxiety are identified pharmacological and non-pharmacological. Pharmacological interventions include sedatives and anti-anxiety drugs. Midazolam, diazepam, ketamine, and fentanyl are the most common anxiolytics (28). As pharmacological treatments have adverse effects such as breathing problems, drowsiness, interfering with anesthetic drugs, and prolonged recovery, non-pharmacological interventions are becoming more commonly used. It is reported that non-pharmacological interventions are more commonly used by anesthesiologists compared to pharmacologic ones in both pediatric and adult anesthesia procedures. For example, in a survey from Korean anesthesiologists, 46.3% preferred non-pharmacological interventions compared to 39.0% preferring medications and 14.6% of no preference for pediatric anesthesia (29). In another report from the UK 95% of anesthesiologists reported the use of communication with the patient and reassurance as their most popular method to reduce preoperative anxiety in the adult population (30).

**Figure 1:**
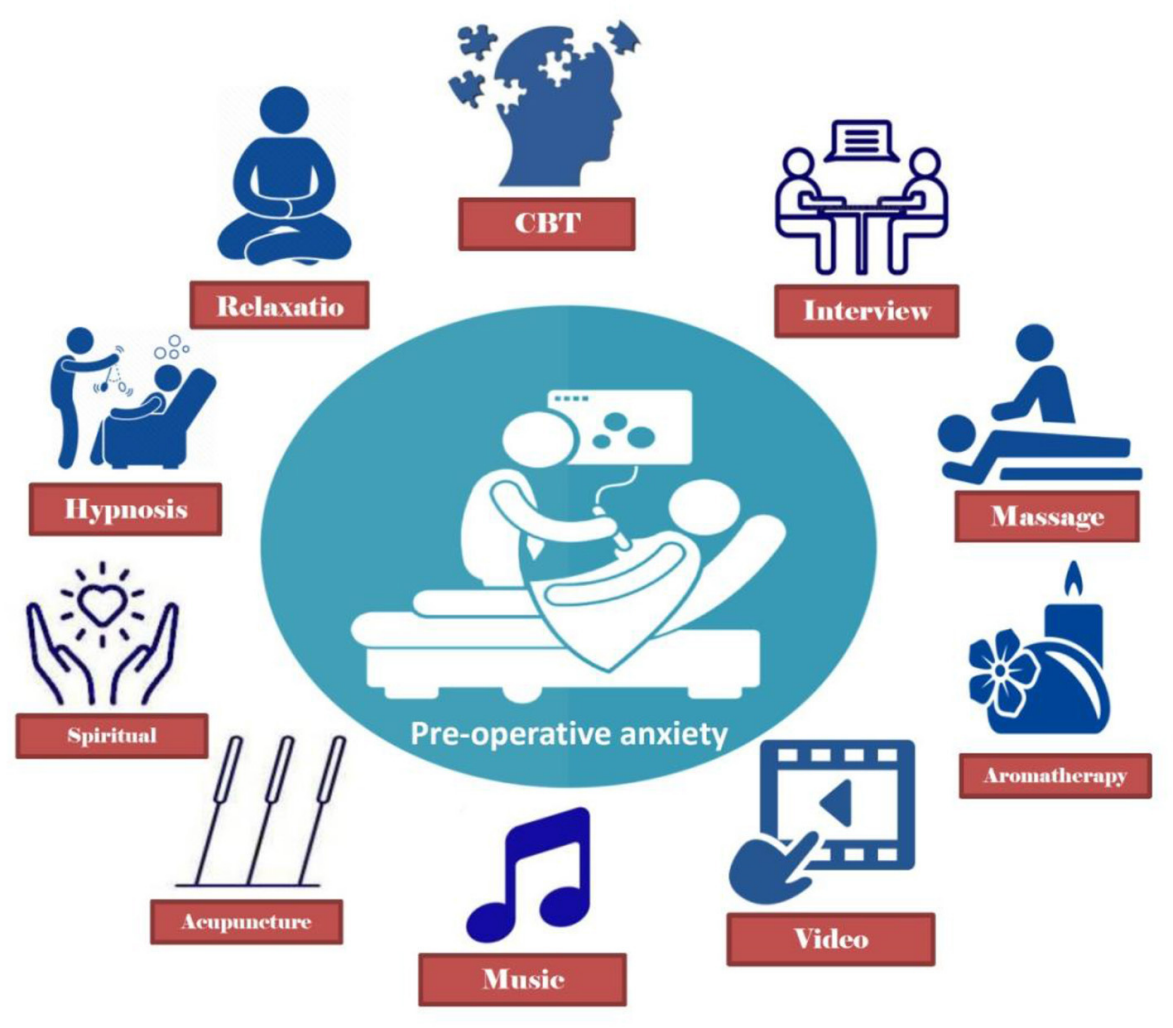
Non-pharmacologic approaches in preoperative anxiety.

Non-pharmacological interventions include, but are not limited to, interviews with patients performed by healthcare providers, communicating strategies, religious or spiritual activity, music, visits from relatives, acupuncture, various distraction, and patient education (Figure 1) (31–44). Various clinical studies are conducted to evaluate the efficacy of these non-pharmacological interventions (Table 1). In this study, the most popular non-pharmacological approaches to preoperative anxiety are reviewed focusing on more recent evidence provided by clinical studies.

**Table 1:** Clinical studies on cognitive behavioral therapy (CBT), guided imagery relaxation and hypnosis in preoperative anxiety.

**References**	**Title**	**Intervention**	**Control**	**Population**	**Surgery**	**Type of study**	**Conclusion**
Dao et al. (45)	Randomized controlled trial of brief cognitive behavioral intervention for depression and anxiety symptoms preoperatively in patients undergoing coronary artery bypass graft surgery	CBT	Standard pre-operative care	100	Coronary artery bypass graft surgery	RCT	CBT targeting preoperative depression and anxiety is both feasible and acceptable for patients undergoing CABG surgery.
Gade et al. (46)	The Impact of a preoperative cognitive behavioral therapy (CBT) on dysfunctional eating behaviors, affective symptoms and body weight 1 year after bariatric surgery: a randomized controlled trial	CBT	Nutritional support and education	80	Bariatric Surgery	RCT	The 10-week CBT intervention showed beneficial effects preoperatively.
Cassin et al. (47)	A pilot randomized controlled trial of telephone-based cognitive behavioral therapy for preoperative bariatric surgery patients	CBT	Standard pre-operative care	47	bariatric surgery	RCT	Tele-CBT holds promise as a brief intervention for improving eating psychopathology and depression in bariatric surgery candidates.
Rajeswari et al. (48)	Effectiveness of cognitive behavioral play therapy and audiovisual distraction for management of preoperative anxiety in children	I—CBT II—audiovisual	Tell-show-do technique	45 children	dental	RCT	Active distraction with cognitive behavioral play therapy is found to be more effective in reducing the preoperative anxiety in children compared to audiovisual distraction and tell-show-do technique.
Birch et al. (49)	No effect of cognitive behavioral patient education for patients with pain catastrophizing before total knee arthroplasty: a randomized controlled trial	CBT	Standard pre-operative care	60	Total knee arthroplasty	RCT	We found no difference in the primary outcome measure, VAS during activity, between the 2 groups but both groups had large reductions over time.
Felix et al. (50)	Guided imagery relaxation therapy on preoperative anxiety: a randomized clinical trial	Guided imagery relaxation	Standard care	24	Video-laparoscopic bariatric surgery	RCT	Guided imagery relaxation therapy is an effective nursing intervention for the reduction of state anxiety and blood cortisol levels in the preoperative period in patients undergoing video-laparoscopic bariatric surgery.
Vagnoli et al. (51)	Relaxation-guided imagery reduces perioperative anxiety and pain in children: a randomized study	Guided imagery relaxation	Standard care	60 children	Minor surgery	RCT	Relaxation-guided imagery reduces preoperative anxiety and postoperative pain in children.
Haipin et al. (52)	Guided imagery in cardiac surgery.	Guided imagery relaxation	Without guided imagery	711	Cardiac surgery	RCT	The guided imagery patient group experienced significantly decreased LOS compared to the control group, thereby lowering hospital costs.
Charette et al. (53)	Guided imagery for adolescent post-spinal fusion pain management: a pilot study	Guided imagery relaxation	Standard care	40	Orthopedic surgery for adolescent idiopathic scoliosis (spinal fusion)	RCT	Addition of a guided imagery and relaxation exercise DVD for home use was more effective than standard care alone for postoperative pain.
Kekecs et al. (54)	Effects of patient education and therapeutic suggestions on cataract surgery patients: a randomized controlled clinical trial	Guided imagery relaxation	Standard care	84	Cataract surgery	RCT	Preoperative information combined with positive suggestions and anxiety management techniques might reduce patient anxiety in the perioperative period of cataract surgery.
Manyande et al. (55)	Preoperative rehearsal of active coping imagery influences subjective and hormonal responses to abdominal surgery	Imagery intervention	Control received background information about the hospital	51	Abdominal surgery patients	RCT	State-anxiety was similar in each group, but imagery patients experienced less postoperative pain than did the controls, were less distressed by it, felt that they coped with it better, and requested less analgesia. Hormone levels measured in peripheral venous blood did not differ on the afternoon of admission, before preparation. Cortisol levels were, however, lower in imagery patients than in controls immediately before and after surgery. Noradrenaline levels were greater on these occasions in imagery patients than controls.
Amraoui et al. (56)	Effects of a hypnosis session before general anesthesia on postoperative outcomes in patients who underwent minor breast cancer surgery: the HYPNOSEIN randomized clinical trial	Hypnosis	Standard care	150 women	Minor breast cancer surgery	RCT	No benefit of hypnosis was found on postoperative breast pain; however, hypnosis seems to have other benefits regarding fatigue, anxiety, and patient satisfaction.
Saadat et al. (57)	Hypnosis reduces preoperative anxiety in adult patients.	Hypnosis	Standard care	50	Ambulatory surgical	RCT	Patients in the hypnosis group were significantly less anxious post-intervention as compared with patients in the attention-control group and the control group.
Duparc-Alegria et al. (58)	Assessment of a short hypnosis in a pediatric operating room in reducing postoperative pain and anxiety: a randomized study	Hypnosis	Standard care	120 children	Major orthopedic surgery.	RCT	This randomized study on a short hypnosis session performed in the operating room prior to a major surgery showed no difference in postoperative anxiety and pain levels. The decrease in anxiety and pain levels may be due to the addition of nurse pre-operative interviews and optimisation in communication in the operating room.
Hermes et al. (59)	Evaluation of intraoperative standardized hypnosis with the state-trait anxiety inventory.	Hypnosis	Standard care	50	Dental surgery	RCT	Hypnosis reduces intraoperative anxiety of oral and maxillofacial patients significantly.
Calipel et al. (60)	Premedication in children: hypnosis vs. midazolam.	Hypnosis	Oral midazolam	50 children	Mixed surgery	RCT	Hypnosis seems effective as premedication in children scheduled for surgery.
Huet et al. (61)	Hypnosis and dental anesthesia in children: a prospective controlled study	Hypnosis	Standard care	30 children	Dental surgery	RCT	Hypnosis may be effective in reducing anxiety and pain in children receiving dental anesthesia.
Ashton et al. (62)	Self-hypnosis reduces anxiety following coronary artery bypass surgery. A prospective, randomized trial	Self-hypnosis	No therapy	32	Coronary artery bypass surgery	RCT	This study demonstrates the beneficial effects self-hypnosis relaxation techniques on patients undergoing coronary artery bypass surgery.
Schnur et al. (63)	Hypnosis decreases presurgical distress in excisional breast biopsy patients	Hypnosis	Presurgery attention control session	90 Women	Excisional breast biopsy	RCT	The study results indicate that a brief pre-surgery hypnosis intervention can be an effective means of controlling pre-surgical distress in women awaiting diagnostic breast cancer surgery.

## Methods

This study was performed as a narrative literature review, aimed to comprehensively gather and summarize the available information in the scientific literature on the non-pharmacologic treatments for preoperative anxiety. The databases of PubMed, Scopus, and web of knowledge, as well as google scholar search engine, were searched for the relevant articles. The searching terms included “preoperative anxiety,” “treatment,” and “trial” from inception to 2020. All studies reported outcomes for non-pharmacologic treatments are reviewed. This study reviewed the latest clinical trials of 10 types of non-pharmacological interventions. The used key terms of treatments, interventions, approaches, and therapy used as synonyms through the text and means the mentioned 10 types of non-pharmacological l interventions aiming the reduction of preoperative anxiety. We will review the clinical evidence about the interview, conversation and communication strategies, cognitive-behavioral therapy, spiritual/religious interventions, music therapy, pre-operation preparation video, aromatherapy, massage, meditation, and guided imagery relaxation therapy, hypnosis, and acupuncture in the treatment of the preoperative anxiety. Animal, and *in-vitro* studies as well as clinical studies on drug therapy for preoperative anxiety were excluded. Two reviewers, independently, checked the studies for eligibility and extracted data from each study. For each study type of non-pharmacologic intervention, type of intervention in control group, sample size, population, type of surgery, and results were extracted. After that, we have discussed the availability, limitations, acceptability, needed education and equipment, acceptability and popularity of these interventions.

## Interview, Conversation, and Communication Strategies

Interview and communication strategies are considered the most common strategy used by anesthesiologists for controlling preoperative anxiety in some studies (30). Different studies have evaluated the efficacy of these strategies in reducing preoperative anxiety. A report evaluating 230 patients undergoing breast and abdominal surgeries by State-Trait Anxiety Inventory (STAI) regarding their preoperative anxiety showed that surgeon communication with the patients and their communication abilities was associated with reduced anxiety scale (64). They have used predefined question prompt lists for the consultation session held 1-3 weeks before the surgical schedule. Another study on a structured communication between patients and anesthesiologists showed reduced anxiety and fear of anesthesia, particularly in younger patients compared to standard interview techniques (65). Notably, the structured interview had a significantly shorter duration compared to the routine sessions. Evidence also supports the positive effect of preoperative patient-doctor communication on pre-operative anxiety (66, 67).

## Cognitive-Behavioral Therapy

Cognitive Behavioral Therapy (CBT) is defined as science-based behavioral and cognitive interventions. Behavioral interventions aim to increase adaptive behaviors and decrease maladaptive ones. Cognitive interventions change abnormal beliefs, self-statements, and cognitions. CBT is the gold standard psychotherapeutic treatment of anxiety disorders (68–71). Exposure and cognitive therapy are the most commonly used CBT methods. Imaginal*, in vivo* (in real life), and interoceptive are three forms of exposure. Cognitive therapy is based on changing the distorted thoughts by using some techniques such as recognizing inaccurate thinking, controlling automatic thoughts, and changing abnormal thoughts (72).

Catastrophizing (such as magnification, rumination, and helplessness), anxiety, and depression are associated with increased postoperative pain (73). Proper evaluation and identifications of these factors in the patient in the perioperative period may help reduce anxiety and promote recovery (74). For example, the modified Yale Preoperative Anxiety Scale (mYPAS) and State-Trait Anxiety Inventory for Children (STAIC) is reported as a significant predictor of anxiety in children (75).

Perioperative Pain Self-management (PePS) intervention based on principles of CBT is a feasible intervention for preventing chronic pain and long-term opioid use (76). A brief Managing Anxiety and Depression using Education and Skills (MADES) intervention (a type of CBT) before coronary artery bypass graft surgery had several beneficial effects in the intervention group compared to usual treatment in control (45). This CBT improved depression and anxiety symptoms and quality of life (45). Moreover, it reduces the length of hospital stay (45) (Table 1).

A randomized controlled trial showed that a 10 week CBT intervention before bariatric surgery, significantly reduced the pre-operative anxiety and depression symptoms (46). Moreover, telephone-based cognitive behavioral therapy (Tele-CBT) improved depression and psychopathology of eating in candidates of bariatric surgery (47). To reduce preoperative anxiety in children, active distraction with cognitive-behavioral play therapy is more effective than the tell-show-do technique, and audiovisual distraction (48). The CBT-based pain education was not more effective than usual care after total knee arthroplasty in reducing pain and improving physical activity (49).

## Spiritual/Religious Interventions

Multiple studies have evaluated the association between religiousness and anxiety with different results in varying communities and cultures (77–79). It is shown that religiousness may be negatively correlated with the level of preoperative anxiety (36, 80). It is also shown that preoperative spiritual/religious training can reduce anxiety in Muslim patients undergoing coronary artery bypass grafting (81). The intervention consists of 5 sessions of 45–60 min in 5 consecutive days presenting relevant Islamic supplication (Zikr) and the holy Quran verses based on Richards and Bergin's (2000) spiritual therapy technique.

## Music Therapy

Music listening triggers the parasympathetic nervous system and reduces sympathetic nervous activity (82). These changes reduce anxiety and help patients to become more relaxed emotionally and physically (82). Moreover, music can distract patients from pain and anxiety (83). Headphones also mask the environmental annoying noises. Heart rate, blood pressure, and respiratory rate may be regulated by listening to music (41, 84). Music is a cheap, safe, non-invasive, and effective non-pharmacological intervention (85). Also, music can reduce the doses of required anesthesia as two clinical trials showed that in patients who listened to favorite music lower doses of propofol (for sedation) and alfentanil were used compared to the control group (86). Music exposure in the preoperative period reduced self-reported anxiety before, during, and after cataract surgery (87). In addition, systolic blood pressure after surgery was significantly lower in patients who had music exposure before cataract surgery than patients of the control group (87). Listening to the favorite music preoperatively before elective inguinal hernia surgery reduced postoperative State-Trait Anxiety Inventory form 1 (STAI-1) score and improved postoperative patient satisfaction in the music group compared to the control group. Although preoperative STAI-1, STAI-2 scores and numeric rating scale (NRS) were similar between the groups (88). The median of the Hospital Anxiety and Depression Scale (HADS) reduced significantly from 7 to 2 after using the music in women who were undergoing elective minor gynecological surgeries (89).

A clinical trial study compared the efficacy of three genres of music on dental surgery anxiety and concluded that classical Western music significantly was effective compared to Turkish music and soft rock music (90) (Table 2). In a randomized clinical trial, outpatient surgery children were randomized into three groups; music, midazolam, and control. Music therapy didn't relieve anxiety during anesthesia induction (91).

**Table 2:** Clinical studies on the efficacy of preoperative music and video in preoperative anxiety.

**References**	**Title**	**Intervention**	**Control**	**Population**	**Surgery**	**Type of study**	**Conclusion**
Marc et al. (86)	The sedative and analgesic sparing effect of music	Music	No-music	78	Urologic procedures	RCT	Use of intraoperative music in awake patients decreases patient-controlled sedative and analgesic requirements.
Muddana et al. (87)	Preoperative and perioperative music to reduce anxiety during first-time phacoemulsification cataract surgery in the high-volume setting: randomized controlled trial.	Music	No-music	165	Phacoemulsification cataract surgery	RCT	Marked reductions in self-reported anxiety before, during, and after surgery when exposed to music and a significant decrease in postoperative blood pressure.
Akelma et al. (88)	Effect of favorite music on postoperative anxiety and pain.	Music	Standard pre-operative care	117	Elective inguinal hernia surgery	RCT	Listening to patient-preferred favorite music preoperatively reduced anxiety, regulated hemodynamic parameters, and improved postoperative patient satisfaction. Reduced anxiety was not associated with reduced pain.
Tan et al. (89)	The effect of perioperative music listening on patient satisfaction	Music	-	83 Women	Elective minor gynecological surgeries	Quasiexperimental study	Perioperative music listening improved patient satisfaction and can reduce patient anxiety and depression.
Kupeli and Gülnahar (90)	Comparing different music genres in decreasing dental anxiety in young adults who underwent third molar surgery in turkey: randomized controlled trial.	Music	No music	80	Third molar surgery	RCT	Classical Western music that was started in the preoperative period and continued until the end of the operation significantly reduced the anxiety.
Kain et al. (91)	Interactive music therapy as a treatment for preoperative anxiety in children: a randomized controlled trial.	Music	Standard pre-operative care	123 Children	Outpatient surgery	RCT	Music therapy may be helpful on separation and entrance to the OR, depending on the therapist. However, music therapy does not appear to relieve anxiety during the induction of anesthesia.
Nguyen et al. (92)	A prospective randomized study on efficacy of music for decreasing preoperative anxiety in children.	Music	No music	150 Children	Children undergoing general anesthesia	RCT	Use of music medicine in the operating room does not show efficacy to reduce anxiety in children based on heart rate changes.
Drzymalski et al. (93)	The effect of patient-selected or preselected music on anxiety during cesarean delivery: a randomized controlled trial	I- patient-selected music II- preselected music	No music	150 Women	Elective cesarean delivery	RCT	Mozart music results in lower anxiety prior to cesarean delivery, but patient-selected Pandora music does not.
Kühlmann et al. (94)	Music interventions in pediatric surgery (the music under surgery in children study): a randomized clinical tria	I-preoperative music intervention II-pre- and intraoperative music	Music intervention (control)	432 children	Orchidopexy, hypospadias, or inguinal hernia repair	RCT	Music interventions do not seem to benefit all young infants undergoing surgery.
Pinto and Hollandsworth (95)	Using videotape modeling to prepare children psychologically for surgery: Influence of parents and costs versus benefits of providing preparation services	Video	No-videotape	60 children	1st-Time elective surgery		Parents who saw the tape or whose children viewed the videotape without them exhibited less arousal prior to the operation than parents who did not and whose children did not view the videotape preparation.
Karabulut and Arikan (96)	The effect of different training programs applied prior to surgical operation on anxiety levels	Video booklet	Standard pre-operative care	90 children	Inguinal hernia	A semi-experimental	Training with the booklet and video was found out to decrease the state loss levels of mothers and children before and after the operation.
Helms (97)	Video education to improve preoperative anxiety in the bariatric surgical patient: a quality improvement project	Video	Preoperative education with the current process of written and verbal instructions	60	Bariatric surgery	quasi-experimental design using a pre-post survey	Addition of an audiovisual component in the form of an informational tour of the perioperative division is an effective method to reduce perceived preoperative anxiety in patients having bariatric surgery.
Lee et al. (98)	Cartoon distraction alleviates anxiety in children during induction of anesthesia	Toy Animated cartoon	Standard pre-operative care	130 Children	Mixed surgery	RCT	Allowing the viewing of animated cartoons by pediatric surgical patients is a very effective method to alleviate preoperative anxiety.
Durst (99)	Preoperative teaching videotape: the effect on children's behavior	Video	Usual preoperative teaching	30 Children	Elective same day surgery	RCT	No significant difference between the control and experimental groups.
Luck et al. (100)	Effects of video information on precolonoscopy anxiety and knowledge: a randomized trial.	Video	No video	198	Colonoscopy	RCT	An information video increases knowledge and decreases anxiety in patients preparing for colonoscopy.
Ayral et al. (101)	Effects of video information on preoperative anxiety level and tolerability of joint lavage in knee osteoarthritis	Video	No video	112	joint lavage	RCT	Preoperative anxiety was lower by half for patients who had viewed the video.
Jlala et al. (102)	Effect of preoperative multimedia information on perioperative anxiety in patients undergoing procedures under regional anesthesia	Video	No video	110	Upper or lower limb surgery	RCT	Preoperative multimedia information reduces the anxiety of patients undergoing surgery under regional anesthesia.
Noben et al. (103)	A virtual reality video to improve information provision and reduce anxiety before cesarean delivery: randomized controlled trial	Video	Standard pre-operative care	97 women	Cesarean delivery	RCT	This study showed that VR does not lead to a decrease in preoperative anxiety.
Wakimizu et al. (104)	A randomized controlled trial of an at-home preparation programme for Japanese preschool children: effects on children's and caregivers' anxiety associated with surgery	Video booklet	Patient-educational video with other patients prior to hospitalization	161 children	Herniorrhaphy	RCT	A specially designed at-home preparation programme as an outpatient care is effective to encourage parent–child verbal interaction concerning surgery and reduce both children and caregivers' anxiety associated with surgery.
Melamed and Siegel (105)	Reduction of anxiety in children facing hospitalization and surgery by use of filmed modeling	Video	Unrelated control film	60 Children	Elective surgeries	RCT	Revealed a significant reduction of preoperative (night before) and postoperative (3–4 wkpostsurgery examination) fear arousal in the experimental as compared to the control film group.

A prospective randomized study showed no efficacy of music used in reducing the preoperative anxiety of children based on heart rate changes (92). Although preselected music caused a reduction of anxiety before cesarean delivery and postoperative pain, the patient-selected one did not. Total satisfaction scores of patients and postoperative anxiety were not different among the music and control groups (93). Among young infants, preoperative and intraoperative music interventions were not effective on preoperative anxiety based on the COMFORT-Behavior scale and physiological measurements such as blood pressure and heart rate (94).

## Pre-Operation Preparation Video

Audiovisual (AV) programs can reduce anxiety and improve coping skills and patients' knowledge (106). Besides, they are used as active (e.g., Interactive games) and passive (e.g., preoperative preparation videos) distraction tools (107). Computers and other technologies (such as video glasses and smartphone applications) are used in this intervention (108, 109). The mechanism of this intervention is based on the interaction between situational anxiety, information retention, and memory (100). The most common type of AV interventions is preoperative (pre-op) preparation videos (99).

Compared with the control group, preoperative anxiety was lower among children who watched peer-modeling pre-op preparation videotape 1 hbefore admission (95) (Table 2). Using 12-min pre-op Video Compact Disc training 48 h before surgery was more effective than pre-op booklet training in children who planned to undergo inguinal hernia surgery (96). Among patients of bariatric surgery, adding audiovisual (film) preoperative information to traditional instructions reduced preoperative anxiety (97).

Watching favorite animated cartoons before anesthesia induction had the lowest modified Yale Preoperative Anxiety Scale (mYPAS) compared with playing with toy and control group in children (98). In contrast, a study showed no significant difference in behaviors related to anxiety between peer-modeling of a pre-op preparation video watching and preoperative teaching (99). Among patients of colonoscopy control and intervention groups were randomly selected. Watching informative videos before colonoscopy significantly reduced anxiety based on Spielberger state anxiety inventory (STAI) in the intervention group before the colonoscopy (100). A clinical trial study among knee osteoarthritis patients reported that patients who watched video information before joint lavage had a lower level of preoperative anxiety and more tolerability (101). In a study, 110 patients of upper and lower limb surgery by regional anesthesia were randomized into study and control groups. Patients in the study group received preoperative multimedia information and they were less anxious than the control group (102). A randomized controlled trial among women who were a candidate for elective cesarean delivery reported that virtual reality information Video did not reduce preoperative anxiety compared to standard preoperative information techniques (103).

Moreover, the efficacy of the combination of video watching with other interventions is evaluated. Using peer-modeling pre-op preparation video combined with an information booklet (104) and watching a generic film (105) was significantly more effective than using video alone.

## Aromatherapy

Some essential oils which are concentrated essences extracted from aromatic plants may have a physiological or pharmacological effect. They can be used via different modes like massage, inhalation, skin absorption, ingestion, and bath (110, 111). Pain control is among the most common indication of essential oils (112). The therapeutic use of these oils is called aromatherapy which is known as a modality of complementary and alternate therapy (113–115). Aromatherapy has been used for thousands of years ago in Egypt and India (111).

Till now, more than 40 types of plant extracts are used for aromatherapy. Lavender oil, rose oil, and citrus species oils are the most commonly used (116–118). Around the world, Aromatherapy is used broadly by nurses as complementary and alternative medicines because it is applied easily and does not need any licensed experts, equipment, and patient involvement (119). Aromatherapy is used for symptom therapy of preoperative anxiety, nausea, vomiting, critical care, wellbeing, anxiety, depression, stress, insomnia, pain, dementia, and oncology in inpatient and outpatient settings (120).

A single-center prospective randomized placebo-controlled trial reported that lavender reduced preoperative anxiety among cataract surgery patients (121) (Table 3). In a study of 30 women undergoing breast surgery a significant reduction of preoperative anxiety was recorded after using a sustained-release lavender oil aromatherapy skin patch (122). Using the inhaled lavender oil in elderly men scheduled for benign prostate hyperplasia (BPH) surgery showed a significant decrease in anxiety and respiration and an increase of oxygen saturation compared to the control group (123). Massage with 5% lavender oil quality in patients with colorectal surgery reduced the anxiety level and increased sleep quality in the preoperative period (124). On the other hand, preoperative inhalation of lavender oil (0.1-mL and 0.3-mL diffused in 120 mL of water) did not have an anxiolytic effect among orthognathic surgery (bilateral sagittal split, Le Fort I, and bimaxillary osteotomies) candidate (125). Moreover, another controlled prospective study in patients who were scheduled for colonoscopy or esophagogastroduodenoscopy showed no beneficial effect of lavender use on preoperative anxiety although that was pleasant to patients (126). Oral use of Citrus aurantium blossom reduced preoperative anxiety in minor operation candidates compared to the control group (127).

**Table 3:** Clinical studies on the efficacy of aromatherapy and massage in preoperative anxiety.

**References**	**Title**	**Intervention**	**Control**	**Population**	**Surgery**	**Type of study**	**Conclusion**
Stanley et al. (121)	Randomized prospective placebo-controlled study of the effects of lavender aromatherapy on preoperative anxiety in cataract surgery patients	Lavender aromatherap	Grape seed oil	75	Cataract surgery	RCT	Lavender aromatherapy reduced anxiety in preoperative cataract surgery patients.
Jaruzel et al. (122)	Aromatherapy for preoperative anxiety: a pilot study	Lavender Aromatherap	Standard pre-operative care	75 Women	Breast surgery	Observational	Use of aromatherapy is beneficial in reducing anxiety experienced by females undergoing breast surgery.
Genc and Saritas (123)	The effects of lavender oil on the anxiety and vital signs of benign prostatic hyperplasia patients in preoperative period	Lavender Aromatherap	Standard pre-operative care	110 Men	BPH surgery	Quasi-experimental	The findings showed that lavender oil inhalation reduced anxiety levels and had effects on the vital signs of BPH patients in their preoperative period.
Ayik and Özden (124)	The effects of preoperative aromatherapy massage on anxiety and sleep quality of colorectal surgery patients: A randomized controlled study	Lavender Aromatherap massage	Standard pre-operative care	80	Colorectal surgery patients	RCT	Aromatherapy massage with lavender oil increased the sleep quality and reduced the level of anxiety in patients with colorectal surgery in the preoperative period.
Bozkurt and Vural (125)	Effect of lavender oil inhalation on reducing presurgical anxiety in orthognathic surgery patients	Lavender aromatherap	No oil	90	Orthognathic surgery	RCT	The results of this study suggested that 1 h of pre-surgical inhalation of 0.1-mL and 0.3-mL lavender oil diffusions in 120 mL of water did not have an anxiolytic effect on patients undergoing orthognathic surgery.
Muzzarelli et al. (126)	Aromatherapy and reducing preprocedural anxiety: a controlled prospective study. Gastroenterology nursing	Lavender aromatherap	Inert oil (placebo)	118	Colonoscopy or esophagogastroduodenoscopy	RCT	This study did not show aromatherapy to be effective based on statistical analysis, patients did generally report the lavender scent to be pleasant.
Akhlaghi et al. (127)	Citrus aurantium blossom and preoperative anxiety	Citrus aurantium Aromatherapy	Saline solution (placebo)	60	Minor operation	RCT	Citrus aurantium blossom may be effective in terms of reduction in preoperative anxiety before minor operation.
Dagli et al. (128)	The effects of aromatherapy using rose oil (Rosa damascena Mill.) on preoperative anxiety: a prospective randomized clinical trial	Rose oil aromatherapy	Standard care	99	Septorhinoplasty/rhinoplasty.	RCT	The application of rose oil aromatherapy by inhalation reduced the scores of preoperative anxiety of patients undergoing septorhinoplasty/rhinoplasty.
Fazlollahpour-Rokni et al. (110)	The effect of inhalation aromatherapy with rose essential oil on the anxiety of patients undergoing coronary artery bypass graft surgery.	Rose oil Aromatherapy	Standard care	66	Coronary artery bypass graft surgery.	RCT	Inhalation aromatherapy with rose essential oil could not significantly reduce anxiety in CABG patients.
Kim et al. (129)	The effect of 1, 8-cineole inhalation on preoperative anxiety: a randomized clinical trial.	Limonene, 1,8-cineole, or eucalyptus oil, Aromatherapy	Almond oil (placebo)	62	Selective nerve root block (SNRB).	RCT	Inhalation of 1,8-cineole may be used to relieve anxiety before, during, and after various operations, in addition to SNRB.
Pasyar et al. (130)	The effect of bergamot orange essence on anxiety, salivary cortisol, and alpha amylase in patients prior to laparoscopic cholecystectomy: a controlled trial	Bergamot orange Aromatherapy	Grape seed oil (placebo)	60	Laparoscopic cholecystectomy	RCT	Bergamot orange essence decreased anxiety and salivary alpha amylase level.
Li et al. (131)	Benefits of hand massage on anxiety in preoperative outpatient: a quasi-experimental study with pre-and post-tests.	Massage	Rest	138	Mixed surgery	Quasi-experimental	After receiving a 15-min non-therapeutic hand massage, patients experienced reduced anxiety levels and increased satisfaction.
Farahani et al. (132)	Effects of extremity massage on preoperative anxiety: a three-arm randomized controlled clinical trial on phacoemulsification candidates.	Massage	Placebo	90 Women	Phacoemulsification cataract surgery	RCT	Application of hand or foot massage seems to be effective in managing anxiety in patients waiting for phacoemulsification cataract surgery.
Brand et al. (133)	The effect of hand massage on preoperative anxiety in ambulatory surgery patients	Massage	Customary nursing care	86	Ambulatory surgery	Quasi-experimental	Hand massage reduces anxiety for patients awaiting ambulatory surgery and outpatient procedures
Mohammadpourhodki et al. (134)	Evaluating the effect of massage based on slow stroke back massage on the anxiety of candidates for cataract surgery.	Massage	Standard care	60	Cataract surgery.	Quasi-experimental	Slow-stroke-back massage, significantly reduces anxiety in patients who are candidates for cataract surgery.
Peng et al. (135)	Effects of massage on the anxiety of patients receiving percutaneous coronary intervention	Massage	Standard care	117	Percutaneous coronary intervention	RCT	Massage treatments reduced the emergency response and level of anxiety of cardiovascular patients before PCI. The post-intervention blood pressure, heart rate, and pain score of the intervention group were significantly better than those of the control group
Rosen et al. (136)	Massage for perioperative pain and anxiety in placement of vascular access devices	Massage	Usual care with structured attention	60	Surgical placement of vascular access devices	RCT	Massage therapy participants had a statistically significant, greater reduction in anxiety after the first intervention compared with individuals receiving structured attention.
McRee et al. (137)	Using massage and music therapy to improve postoperative outcomes	I-massage with music therapy II- massage only III- music therapy only	Standard care	52	Mixed surgery	Quasi-experimental	Ostoperative anxiety levels were significantly lower and postoperative prolactin levels were significantly higher for all groups.

Inhalation of rose oil before septorhinoplasty/rhinoplasty surgeries was effective in decreasing preoperative anxiety in a prospective randomized clinical trial (128). In contrast, a single-blind randomized clinical trial showed that inhalation of three drops of 4% rose essential oil for 10 min in one night and 1 h before coronary artery bypass graft (CABG) was not significantly effective in reducing preoperative anxiety (110). Inhalation of 1,8-Cineole (the major component of eucalyptus) in a randomized controlled trial Performed in 62 patients before selective nerve root block (SNRB) showed the efficacy of this type of aromatherapy in anxiety reduction (129). A pre-post-designed clinical study performed on candidates for cholecystectomy showed that bergamot orange essence can help in decreasing anxiety (130).

## Massage

Touching and manipulation of soft tissue for therapeutic goals are named massage (138). It has been used as a therapeutic intervention since thousands of years ago especially in china (139). Hand massage as a non-pharmacological, simple, cheap, and non-invasive nursing intervention can significantly reduce preoperative pain, anxiety, and stress. In addition, it can improve positive feelings like relaxation, calmness, and satisfaction (133, 140–142). No side effects are reported about massage (143). Various mechanisms for the therapeutic effect of massage are explained theoretically. Massage reduces pain by muscle relaxation and enkephalins release (139, 144). The powerful stimulus of massage is conducted faster than pain along nerve pathways to the brain so massage can block pain conduction at the peripheral points (gaits of pain) and relieve pain (139, 145). Another mechanism explains that massage can increase the circulation of soft tissue so irritant substances including lactic acid and inflammatory substances are removed from the tissue. Besides, massage reduces pain sensation by inducing a sense of wellbeing (146).

A pre-and post-test quasi-experimental study (without a control group) reported that one session of 15-min hand massage help in reducing preoperative anxiety levels and increasing satisfaction (Table 3) (131). The massage was provided by Caring Hands massage volunteers from the 7 days Mayo Clinic Volunteer Program (131). A three-arm randomized study compared the efficacy of hand and foot massage with placebo and reported that a one session 5-min massage before cataract surgery significantly reduced anxiety and no significant differences were seen between hand and foot massage based on visual analog scale and physiological indicators (132). The massage was provided by a qualified nursing assistant, who had successfullycompleteda 12-h a workshop on therapeutic applications of classic massage 10 min before the surgery (132). Hand massage also reduced anxiety among patients in the ambulatory surgery setting (133). Slow stroke back massage for 15 min 30 min before surgery significantly reduced anxiety in cataract surgery candidates (134). Massage intervention for 20 min in one session for cardiovascular patients before percutaneous coronary intervention (PCI) reduced anxiety level and emergency response. Moreover, blood pressure, pain score, and heart rate after the operation were lower in comparison to the control group (135). A randomized, controlled trial (RCT) compared massage therapy with usual cares by using the State-Trait Anxiety Inventory (STAI) and 11-point numerical rating scale (0 = no pain to 10 = worst possible pain) among patients with cancer who were scheduled for surgical insertion of the vascular access device and concluded that a 20-min massage therapy before and after operation significantly reduced anxiety (136). A study evaluated the effect of music and massage among three intervention groups and one control group. In one group, patients had one session of 30 min of massage therapy and 30 min of music listening before the operation. In the second group, patients had 30 min of music listening and patients of the third group had 30 min of massage before the operation. Standard care was done for patients in the control group. Then the hemodynamic status (blood pressure and pulse rate), level of serum cortisol, and prolactin and anxiety level (by using STAI-6) were measured. This study showed that preoperative anxiety scores, preoperative and post-operative cortisol levels, and blood pressure were not significantly different among the 4 groups but postoperative anxiety scores were lower in intervention groups compared with the control group. Moreover, the combination of music and massage more effective than using massage or music alone in reducing postoperative anxiety (137).

Massage for pre-operative anxiety needs some equipment like a specific bed and trained personnel. Patients with pain and tenderness in massage site, severely immunocompromised state, pregnancy, bleeding disorder, dermatologic problems, allergy/sensitivity to gloves or massage oil, acute coronary syndrome, neuropathy, or delirium are not suitable candidates for this technique and are excluded in many studies. Another important point about the studies on this technique is that blinding the participant is not possible due to the nature of the intervention.

## Meditation and Guided Imagery Relaxation Therapy

Guided imagery relaxation therapy is a relaxing technique based on the interaction between the brain, mind, body, and behavior. Relaxation means being free from physiological and psychological tension. In this technique, the patient changes negative or stressful feelings by focusing on pleasing images (147). Images can be visual, auditory, tactile, and motor forms (148).

A randomized, triple-blind clinical trial which was done to evaluate the effect of Guided imagery relaxation therapy on preoperative anxiety reported that this intervention significantly reduced anxiety among video-laparoscopic bariatric surgery candidates (50). A randomized study investigated the effect of Guided imagery relaxation therapy in reducing preoperative anxiety and postoperative pain among children and reported that Guided imagery relaxation therapy (Table 1).

Significantly reduced postoperative pain and preoperative anxiety in children (51). Among patients of cardiac surgery, guided imagery relaxation therapy reduced the pain, length of hospital stay, and anxiety (52). A study was conducted among patients of spinal fusion surgery and compared intervention (using a DVD with information and guided imagery/relaxation practices) with the control group. Lower post-operative pain intensity was reported among the intervention group but coping strategies (eating, sleeping, and walking) and State-trait anxiety were not significantly different (53). A randomized controlled clinical trial study concluded that a combination of preoperative information with techniques of anxiety management and positive suggestions reduced perioperative anxiety in cataract surgery patients (54). A study compared 26 imagery patients with 25 controls and reported that by using imagery relaxing therapy, cortisol level was reduced but noradrenaline levels were higher than controls, and the level of State-anxiety was similar in both groups (55). Another report on 100 patients undergoing cataract surgery receiving mediation showed reduced preoperative anxiety measured by the Amsterdam Preoperative Anxiety and Information Scale compared to the control group (149).

## Hypnosis

Since many years ago, hypnosis has been used in surgical processes to reduce the amount of administered analgesics (150). Hypnosis is a modified state of consciousness that is different from normal consciousness and sleep stages.

In hypnosis patients are put in induction of a trance state so acceptable suggestions are delivered to patients. Hypnosis helps patients to improve their performance, perceptual, sensory, and memory abilities (151). The therapeutic effect of hypnosis is based on perception and attention alterations. Hypnotherapists achieve clinical goals such as anxiety, pain, and nausea reduction by distracting attention and modifying perception in patients (152–154). Hypnosis consists of three phases, first phase is induction that helps the patients to be relaxed. In the second phase suggestions (symptom therapies) are delivered to patients. In the third phase, the patient is backed to a normal consciousness state (155). Hypnosis is considered as an adjunctive or primary intervention and is used for managing anxiety, acute or chronic pain especially in children (156–158).

In a trial study consisting of 3 groups, the control, hypnosis, and attention control groups were compared and it was reported that post-intervention anxiety was significantly lower in the hypnosis group (57) (Table 1). A clinical trial was conducted among 150 women who underwent minor breast surgery and reported that hypnosis reduced postoperative anxiety and fatigue score compared to the control group but the level of post-operative breast pain was not significantly different among them (56). A randomized trial in 120 children showed that a short hypnosis session before the operation had no beneficial effect on postoperative pain and anxiety in comparison to the control group (58). Using hypnosis in patients of dental surgery significantly reduced intraoperative anxiety compared to the control group (using only local anesthesia as standard care) (59). A clinical randomized study compared the efficacy of midazolam and hypnosis among children. They assessed anxiety level by using the mYPAS score in four phases (the first phase entering the department, second entering the operation room, third fixing the face mask) and postoperative behavioral changes by using the Post-hospitalization Behavioral Questionnaire (PHBQ) and concluded that the effect of hypnosis was similar to midazolam in reducing preoperative anxiety (60). Among children who had hypnosis therapy at the time of anesthesia, anxiety (anxiety level was assessed by the modified Yale preoperative anxiety scale) was significantly lower compared to the control group (61). In a study, patients who had self-hypnosis experienced more postoperative relaxation and had lesser use of pain medications unlike the control group (62). A 15-min hypnosis therapy before incisional breast biopsy reduced distress before surgery [based on visual analog scales (VAS)] compared to the control group (63).

## Acupuncture

Acupuncture is a traditional treatment that originated from China and spread through the world that uses needling specific points through the patient's skin for therapeutic purposes (159). Acupuncture is increasingly used and investigated for its potential in treating preoperative anxiety (160). A meta-analysis of the 13 published clinical trials, including 439 patients and 595 control participants, evaluating the effect of acupuncture techniques on preoperative anxiety showed the statistically significant superiority of acupuncture compared to placebo or no-treatment groups (160). Studies offered acupuncture sessions lasting between 10 and 30 min; sessions were conducted in operating waiting rooms on the day of surgery using acupuncture needles, balls, and beads in body and/or auricular acupoints (160).

## Discussion

The review of literature on the non-pharmacologic interventions for preoperative anxiety showed a wide range of options evaluated for this indication with promising results. However, there are some concerns about the availability, cost, and required educated personnel to bring these methods into clinical practice. For example CBT, due to the limited number of practitioners, even in developed countries, can be difficult to find. Similar limitations may be present in practicing meditation and guided imagery relaxation therapies. On the other hand applying techniques such as aromatherapy and music is easy without the need for a specially trained therapist and can be more widely recommended and practiced.

Regarding the person who delivers the non-pharmacologic method to the patient, there is not unique practice in different methods and even in the same method among different studies. Interview and communication strategies have mostly been practiced by the surgeon or anesthesiologist of the patient. Cognitive-behavioral therapy is mostly delivered by trained psychologists. The limitations in the access to trained psychologists for practicing CBT led to attempts to turn the technique into a simplified therapy administered by nurses using treatment manuals. However, manualized therapies have limitations, especially facing more complicated patients and the efficacy is more limited compared to the trained psychologists who delivered CBT (161). Hypnosis also suffers similar limitations. Meditation and guided imagery relaxation therapy, massage, and acupuncture of specific points are among the easier techniques which can be more simplified and manualized. These techniques are routinely practiced by different members of the perioperative care team, mostly nurses, passing the short training courses. At the end of the spectrum aromatherapy, preparative video training, and music therapy can be even self-practice by the patients following simple instructions without the need for a specific training course for the delivery of these methods.

Regarding the timing of delivery of non-pharmacologic methods for preoperative anxiety, most studies evaluated the efficacy of these methods the day before or the same day of surgical procedure. However, this time seems to be late because many patients may suffer anxiety from the time scheduled for the surgery which is mostly long before the surgical procedure. It is reported that assessment of patients about the level of preoperative anxiety 1–2 weeks prior to the surgical procedure was more effective in alleviating the anxiety compared to the preoperative visit on the night before surgery (7). So earlier assessment of anxiety is recommended which provides us the opportunity of earlier intervention and referral of patients with a higher level of anxiety for a psychological consultation (7).

This study has some limitations. First of all, this study is just a narrative review of some clinical trials which were conducted to evaluate the efficacy of some of the non-pharmacological interventions for reducing preoperative anxiety. Second, we didn't evaluate the quality of the trials by a specific objective instrument. Beside these limitations the study has important strength which is the first study that gathers the current clinical evidence on non-pharmacologic treatments for anxiety, altogether.

## Implications of the Results for Practice, Policy, and Future Research

As described above, due to the side effects of pharmacological interventions of preoperative anxiety, non-pharmacological interventions are becoming an alternative suggested item. Overall, few side effects are reported about non-pharmacological interventions so they can be used in patients of different ages and types of disease and surgery. As there are some controversies about the efficacy of these interventions in preoperative anxiety, more randomized clinical trials with a larger sample size are needed to evaluate the efficacy of these interventions.

## Author Contributions

XH and YW designed the work, reviewed the literature, and drafted the first version of the manuscript. RW and MA researched the literature, added some parts, and critically revised the article. All authors contributed to the article and approved the submitted version.

## Conflict of Interest

The authors declare that the research was conducted in the absence of any commercial or financial relationships that could be construed as a potential conflict of interest.

## Publisher's Note

All claims expressed in this article are solely those of the authors and do not necessarily represent those of their affiliated organizations, or those of the publisher, the editors and the reviewers. Any product that may be evaluated in this article, or claim that may be made by its manufacturer, is not guaranteed or endorsed by the publisher.
